# Clinical Potential and Current Progress of Dental Pulp Stem Cells for Various Systemic Diseases in Regenerative Medicine: A Concise Review

**DOI:** 10.3390/ijms20051132

**Published:** 2019-03-06

**Authors:** Yoichi Yamada, Sayaka Nakamura-Yamada, Kaoru Kusano, Shunsuke Baba

**Affiliations:** 1 Department of Oral Implantology, Osaka Dental University, 1-5-17 Otemae Chuo-ku, Osaka 540-0008, Japan; yamada-s@cc.osaka-dent.ac.jp (S.N.-Y.); kusano-k@cc.osaka-dent.ac.jp (K.K.); baba-s@cc.osaka-dent.ac.jp (S.B.); 2 Department of Infectious Diseases and Applied Immunology, IMSUT Hospital of The Institute of Medical Science, The University of Tokyo, 4-6-1 Shiroganedai, Minato-ku, Tokyo 108-8639, Japan

**Keywords:** dental pulp stem cells (DPSCs), stem cell therapy, mesenchymal stem cells, systemic disease, clinical application

## Abstract

Dental pulp stem cells (DPSCs) are mesenchymal stem cells (MSCs) that have multipotent differentiation and a self-renewal ability. They have been useful not only for dental diseases, but also for systemic diseases. Extensive studies have suggested that DPSCs are effective for various diseases, such as spinal cord injuries, Parkinson’s disease, Alzheimer’s disease, cerebral ischemia, myocardial infarction, muscular dystrophy, diabetes, liver diseases, eye diseases, immune diseases, and oral diseases. DPSCs have the potential for use in a cell-therapeutic paradigm shift to treat these diseases. It has also been reported that DPSCs have higher regenerative potential than the bone marrow-derived mesenchymal stem cells known as representative MSCs. Therefore, DPSCs have recently gathered much attention. In this review, the therapeutic potential of DPSCs, the latest progress in the pre-clinical study for treatment of these various systemic diseases, and the clinical applications of DPSCs in regenerative medicine, are all summarized. Although challenges, including mechanisms of the effects and establishment of cell processing and transplantation methods for clinical use, still remain, DPSCs could be promising stem cells sources for various clinical applications, because of their easy isolation by a noninvasive procedure without ethical concerns.

## 1. Introduction

Mesenchymal stem cells (MSCs) are multipotent stem cells characterized by self-renewal and multilineage differentiation. They exist in almost all tissues and play a significant role in tissue repair and regeneration [1]. Recently, clinical trials for MSC-mediated therapies have rapidly developed, and there is high potential for using MSCs as a novel therapy for many diseases [1,2,3,4,5,6,7]. The bone marrow-derived mesenchymal stem cells (BMMSCs) are the most widely studied and utilized in clinical settings [1,2,3]. In addition, stem cells can be isolated from various tissues, including oral parts such as alveolar bone, dental pulp, periodontal ligament, dental follicle, apical papilla, oral mucosa, and gingiva [8,9,10,11,12,13,14,15]. These dental stem cells are thought to originate from the cranial neural crest and are referred to as mesenchymal stem cells [16].

Dental pulp stem cells (DPSCs) were the first human dental stem cells isolated from the dental pulp of permanent teeth [8], and stem cells from the deciduous teeth of infants, termed SHED (stem cells from human exfoliated deciduous teeth) have also been isolated [17]. Dental pulp is a connective tissue encapsulated in the mineralized tooth structure that has several vital functions, including the formation of reparative dentin to protect pulp tissue against noxious external stimuli. DPSCs and SHED display an MSC-like character, including the capacity for self-renewal and multilineage differentiation. Because of their easy accessibility, isolation by noninvasive routine clinical procedures, very limited ethical concerns, and high proliferation capacity, DPSCs and SHED are thought to be promising stem cell sources for clinical use.

**Figure 1:**
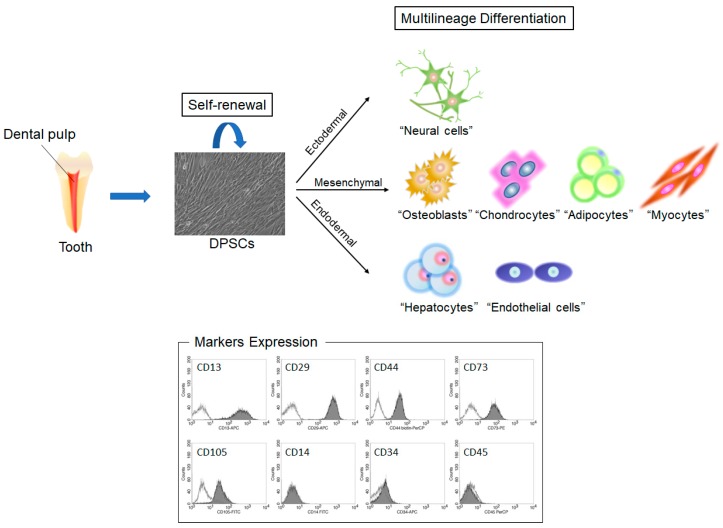
Characteristics of dental pulp stem cells (DPSCs). DPSCs can be obtained from dental pulp tissue and possess self-renewal and multilineage differentiation potential. DPSCs expressed mesenchymal stem cell (MSC) markers resembling those of bone-marrow-derived MSCs (BMMSCs).

In our previous studies, we performed flow cytometry analysis using mesenchymal lineage markers (CD13, CD29, CD44, CD73, and CD105), a monocytic marker (CD14), and hematopoietic lineage markers (CD34, and CD45) to characterize DPSCs. DPSCs are positive for mesenchymal lineage markers and negative for monocytic and hematopoietic lineage markers (Figure 1) [18]. One of the unique characteristics of DPSCs is that they have the potential to differentiate into not only typical mesodermal cell lineages, such as osteogenic, chondrogenic, and adipogenic lineages, but also ectodermal and endodermal cell lineages. We also showed that DPSCs and SHED possess higher proliferation rates than BMMSCs [19]. In vivo studies demonstrated that DPSCs could reconstitute functional dentin/pulp complexes as well as other tissues, such as bone, cementum, blood vessels, and neural tissues [20,21,22,23,24].

**Figure 2:**
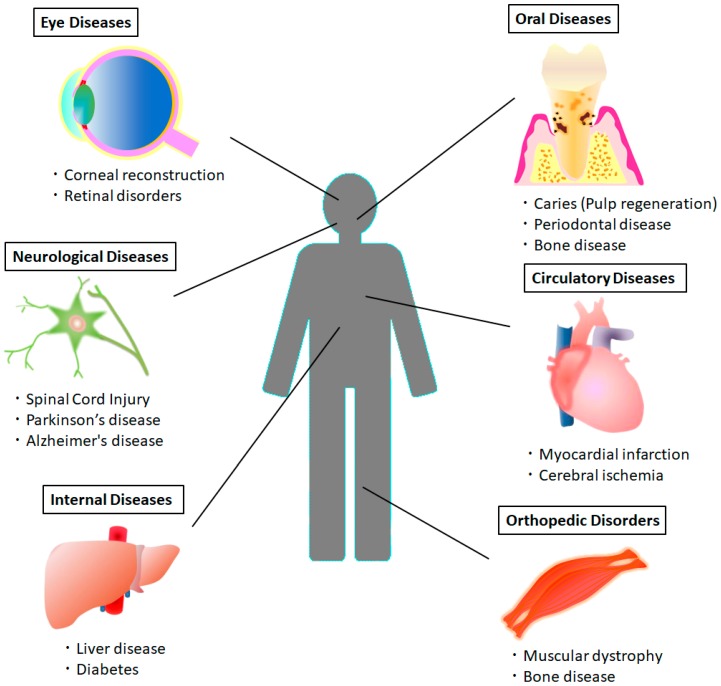
Application of DPSCs for treating various disorders. Transplantation of DPSCs or DPSC- derived differentiated cells is beneficial not only for oral diseases, but also for systemic diseases such as neurological disease, circulatory disease, internal disease, orthopedic disorders, and eye disease.

Recently, DPSCs have been widely studied because they have been reported to be useful not only for dental diseases but also for various systemic diseases (Figure 2). DPSCs have exhibited the potential to differentiate into active neurons, cardiomyocytes, myocytes, melanocytes, and hepatocyte-like cells [23,24,25,26,27,28,29]. Dental pulp tissue is thought to be derived from migrating neural crest cells and DPSCs express neuronal lineage markers such as nestin and βIII-tubulin, as well as neurotrophic factors [16,29,30,31]. DPSCs also have the potential for use in cell-therapeutic paradigms to treat neurological disease [25,32]. Previous reports demonstrated that DPSCs have higher angiogenic, neurogenic, and regenerative potential than BMMSCs [33]. Additionally, the immunomodulatory properties of DPSCs place them as a more useful cell source for cell-based therapy of immune and inflammation-related diseases [4]. DPSCs are promising alternative sources of multipotent MSCs. In the current review, we focus on the latest progress in studying DPSCs for the treatment of systemic diseases and the potential for future studies with clinical applications.

## 2. Therapeutic Potential of DPSCs in Various Systemic Diseases

There is substantial evidence that DPSCs and SHED have great potential to treat various systemic diseases (Figure 2). A summary of publications that indicate multipotent abilities and the various approaches using DPSCs and SHED in animal or in vitro disease models is shown in Table 1.

### 2.1. Spinal Cord Injury (SCI)

Nosrat IV et al. first reported the possibility of using dental pulp to treat spinal cord injuries. They grafted dental pulp tissue into a hemisected spinal cord and showed increases in the number of surviving motoneurons in rats, indicating functional bioactivity of the dental-pulp-derived neurotrophic factors in vivo by rescuing motoneurons [65]. Several studies have reported that DPSCs or SHED transplantation functionally recovered SCI [31,34,35,36]. They showed that the grafted cells migrated in the host spinal cord and differentiated in vitro toward neural cells. Taghipour et al. used neural induced SHED (iSHED) as well as SHED for transplantation to a model of SCI, and animals that received iSHED were in a better state as compared with the SHED group [34]. Neural-induced SHED may be more useful for SCI treatment because they have greater potential for differentiation into oligodendrocytes and/or secretion of neurotrophic factors in the lesioned site of the spinal cord and involvement in recovery from SCI. The therapeutic benefits of transplanted cells in SCI might be due to both cell-autonomous and paracrine effects.

### 2.2. Parkinson’s Disease (PD)

Parkinson’s disease (PD) is a neurodegenerative disorder that is characterized by the loss of dopaminergic neurons. Intracerebral transplantation of dopaminergic neurons or progenitors derived from fetal neural tissues has been proven to be a promising approach for Parkinson’s disease (PD) treatment [37,38]. The therapeutic effect of SHED was studied in Parkinsonian rats [37,38]. The researchers reported that SHED had abilities of dopaminergic differentiation and that transplantation of SHED into the striatum of Parkinsonian rats improved their behavioral disorders. DPSCs possess superior neuronal plasticity toward dopaminergic neurons compared to SHED [66]. Other groups have investigated the neuroprotective effects of DPSCs against MPP+ and rotenone in an in vitro model of Parkinson’s disease [39]. They concluded that dental-pulp-derived neurotrophic factors provide neuroprotection for dopaminergic neurons against MPP+ or rotenone toxicity in vitro.

### 2.3. Alzheimer’s Disease (AD)

Alzheimer’s disease (AD) is an incurable neurodegenerative disease characterized by a decline in cognitive abilities and the appearance of β-amyloid plaques in the brain. Many types of stem cells have been used in Alzheimer’s disease (AD) therapy with some favorable effects [67]. The therapeutic possibility of DPSCs was investigated using in vitro AD models [40,41]. The co-culture of dental pulp cells with hippocampal and mesencephalic neurons treated with amyloid beta peptide or 6-hydroxydopamine (6-OHDA) significantly reduced the toxicity of neurons and increased neuronal viability in an in vitro AD model [40]. They also showed that human dental pulp cells expressed a neuronal phenotype and produced neurotrophic factors such as the nerve growth factor, glial-cell derived neurotrophic factor, brain-derived neurotrophic factor, and bone morphogenetic protein 2. Wang et al. used an okadaic acid (OA)-induced in vitro AD model to examine therapeutically the effect of human DPSCs [41]. They showed that DPSCs caused significant increase in the viability and decrease in apoptosis of the model cells. In addition, DPSCs-treated cells had the morphology of restored neurons, with elongated dendrites, densely arranged microfilaments, and thickened microtubular fibrils. They concluded that repairing effect of DPSCs might be due to the various growth factors secreted by DPSCs.

### 2.4. Cerebral Ischemia

DPSCs have been reported to have potential for the treatment of cerebral ischaemia [42,43,44,45]. Local transplantation of CD31^−^, CD146^−^ side population (SP) DPSCs that were isolated from porcine dental pulp using fluorescent Hoechst dye 33342 caused the functional revascularization of mouse hind limb ischemia [68]. These cells also promoted the migration and differentiation of the endogenous neuronal progenitor cells, induced vasculogenesis, and ameliorated ischemic brain injury after middle cerebral artery occlusion [42]. A comparative study of the therapeutic potential of intravenous transplantation of human DPSCs and human bone marrow-derived MSCs in a rat stroke model indicated that DPSCs showed a greater reduction in infarct volume compared to bone marrow-derived MSCs [44]. These studies showed that DPSCs could migrate and survive within a central nervous system lesion site, offering a suitable therapy for brain injury either through predifferentiation and replacement of lost neurons or through paracrine-mediated supporters of endogenous neuronal survival [30].

### 2.5. Myocardial Infarction

Gandia et al. reported that DPSCs could be useful to repair infarcted myocardium [46]. After injection of DPSCs into an acute myocardial infarction model of rat, cardiac function was improved and infarct size was reduced. This is probably because of their ability to secrete proangiogenic and antiapoptotic factors. Furthermore, conditioned medium of SHED also has a therapeutic effect on acute cardiac injury by suppressing inflammation and apoptosis [69].

### 2.6. Muscular Dystrophy

Duchenne muscular dystrophy (DMD) is one of the most common and severe of the muscular dystrophies that are caused by mutations in a large gene located at Xp21, which encodes the muscle protein dystrophin. Kerkis et al. transplanted SHED into golden retriever muscular dystrophic dogs without immunosuppression and demonstrated that donor SHED can engraft, differentiate, and persist in the host muscle [47]. Although modest dystrophin expression was observed, the clinical benefit was apparent in treated animals. This is due to the immune modulatory effect of stem cells from dental pulp [47]. In another study, human DPSCs that were pre-differentiated toward a myogenic lineage were injected into the gastrocnemius muscles of mdx/SCID mice to test their ability to regenerate dystrophin-expressing muscle fibers within the dystrophic skeletal muscle of mdx/SCID mice [48]. DPSCs engrafted within the host muscle promoted angiogenesis and reduced fibrosis through a paracrine effect, which eventually led to an improvement of the histopathology of the dystrophic muscle [48]. Human dental pulp pluripotent-like stem cells also showed integration in muscular fibers and vessels when engrafted in the skeletal muscle of dystrophic mice [49].

### 2.7. Diabetes

DPSCs reported to be differentiated into a pancreatic cell lineage and could be used for autologous stem cell therapy in diabetes [70]. The same group investigated the therapeutic ability of DPSCs and SHED using streptozotocin-induced diabetic mice. Mice transplanted with macro-capsules containing islet-like cell clusters (ICCs) derived from DPSCs and SHED were restored to normoglycemia [50]. Other researchers have evaluated the therapeutic potential of mouse DPSCs in important complications of diabetes, and have demonstrated that DPSCs reduced pancreatic damage and improved both renal function and painful diabetic neuropathy [51]. The immunomodulatory effect of DPSCs was shown in diabetic rats, and it might play an important role in treatment for diabetic polyneuropathy [52]. Datta et al. compared the beneficial effects of DPSCs’ transplantation by two routes, intravenous or intramuscular, and demonstrated that the intramuscular route with repeated doses was the most effective [53].

### 2.8. Liver Disease

It was reported that DPSCs and SHED could differentiate into hepatocyte-like cells which exhibit a hepatocytic morphology [27,28]. Transplantation of SHED recovered the liver dysfunction of carbon tetrachloride (CCl_4_) -treated mice [54]. The study demonstrated that donor SHED survived to differentiate into human hepatocytes that expressed human-hepatocyte-specific genes and secreted human albumin, urea, and blood urea nitrogen in CCl_4_-injured liver tissues, suggesting that SHED-derived direct-converted hepatocytes might have a therapeutic effect on liver fibrosis. DPSCs that were hepatically differentiated also suppressed liver fibrosis and restored serum levels of alanine transaminase, aspartate transaminase, and ammonia [55,56].

### 2.9. Eye Disease

Recent studies have explored the potential of DPSC-mediated repair of ocular diseases, such as corneal blindness and glaucoma [57,60]. It was reported that DPSCs share similar characteristics with limbal stem cells and have the capability to differentiate into keratocytes [57,71]. Corneal reconstruction occurred with DPSCs in an animal model of limbal stem cell deficiency [58,59]. The loss of retinal neurons, their connections, and supporting glia in ocular degenerative diseases causes permanent blindness, principally because lost photoreceptors and retinal ganglion cells (RGCs) are not replaced and RGC axons fail to regenerate [72]. Recently, DPSCs have been suggested to have the potential to differentiate into RGC-like cells [73]. When DPSCs were delivered into the vitreous of glaucomatous rodent eyes, DPSCs provided significant protection from RGC loss and preserved visual function [30,60].

### 2.10. Immune Disease

MSCs possess potent immunomodulatory functions which render them a potential novel immunotherapeutic tool for a variety of autoimmune and inflammation-related diseases. The major mechanisms may involve the secretion of soluble factors, such as prostaglandin E2 (PGE2), indoleamine 2, 3-dioxygenase (IDO), transforming growth factor-β (TGF-β), and human leukocyte antigen G5 (HLA-G5), and interactions between MSCs and immune cells such as T cells, B cells, macrophages, and dendritic cells [4]. DPSCs and SHED have also been found to possess immunomodulatory functions, similar to BMMSCs. Therefore, they are thought to be promising candidates in cell-based therapies of a variety of immune and inflammation-related diseases [4]. An in vitro study showed that DPSCs inhibited T-cell response more strongly than BMMSCs [74]. SHED had significant effects on inhibiting T helper 17 cells compared to BMMSCs, and SHED transplantation was capable of effectively reversing systemic lupus erythematosus (SLE)-associated disorders in SLE-like mice [61]. DPSCs could inhibit acute allogeneic immune responses by the release of TGF-β as a result of allogeneic stimulation of T lymphocytes [75]. These results provide novel insight for the allogeneic transplantation of DPSCs in future clinical use.

### 2.11. Oral Diseases

DPSCs are also useful for oral diseases such as caries, periodontal disease, and alveolar bone atrophy. Nakashima’s group examined total pulp regeneration using an adult canine model of pulpectomy [62]. They trasnplanted pulp CD105+ stem cells into a root canal with stromal cell-derived factor-1 after pulpentomy. This study demonstrated that the root canal was successfully filled with regenerated pulp, including nerves and vasculature, followed by new dentin formation. Another study examined the usefulness of SHED for pulp/dentin regeneration [63]. They transplanted SHED into full roots from extracted human premolars with Puramatrix^TM^ or recombinant human collagen type I and implanted subcutaneously into immunodeficient mice. The transplantation of SHED generated a pulp-like tissue throughout the extent of the root canal and new dentin generation was also observed. Khorsand et al. demonstrated the potential of DPSCs for periodontal renegeration [64]. Autologous canine DPSCs with Bio-Oss were transplanted into *a* periodontitis model and regeneration of periodontal tissue including cementum, bone, and periodontal ligament was observed. Yamada et al. investigated the ability of bone regeneration by DPSCs or deciduous tooth stem cells [21]. After transplantation of DPSCs or deciduous tooth stem cells with platelet-rich plasma into a canine alveolar bone atrophy model, well-formed mature bone containing neovascularization was observed. In addition, implantation of dental implants into the regenerated bone showed successful osseointegration, indicating the usefulness of DPSCs for the restoration of normal mastication.

## 3. Clinical Application of DPSCs

In contrast to the extensive evidence that has been reported from basic studies, very few clinical studies using DPSCs have been published. Nakashima et al. published a pilot clinical study using mobilized autologous DPSCs for complete pulp regeneration based on preclinical bench studies [76,77]. Five patients with irreversible pulpitis were enrolled and monitored for up to 24 weeks following DPSCs’ transplantation. The authors used a granulocyte colony-stimulating factor (G-CSF)-induced stem cell mobilization method for the enrichment of DPSCs subsets. They demonstrated that DPSC transplantation with G-CSF in an atelocollagen scaffold in pulpectomized teeth was safe and effective. Briefly, the clinical and laboratory evaluations showed no adverse events or toxicity. The electric pulp test (EPT), which is the most commonly used method in clinical practice to determine pulp status, was positive after cell transplantation in four patients. The signal intensity of magnetic resonance imaging (MRI) of the regenerated tissue in the root canal after 24 weeks was similar to that of normal dental pulp, indicating complete pulp regeneration. Another group performed a randomized, controlled clinical trial using human deciduous autologous pulp stem cells for dental pulp regeneration [78]. Patients with pulp necrosis after traumatic dental injuries were enrolled in the clinical trial and 26 patients after DPSC implantation and 10 patients after apexification treatment were examined. 12 months after treatment, regeneration of three-dimensional pulp tissue equipped with blood vessels and sensory nerves were observed in the DPSC implantation group. In addition, the patients with DPSC implantation did not observe any adverse events.

Based on our basic and preclinical studies that showed the usefulness of DPSCs in bone regeneration [21,79,80,81], a clinical protocol was prepared in accordance with the principles of the Declaration of Helsinki and the Japanese guidelines of human stem cell clinical research. After approval by the institutional review boards and the Japanese Ministry of Health, Labor and Welfare, we conducted a pilot clinical trial of bone regeneration. Autologous DPSCs were prepared in a cell processing center according to a standard operating procedure (SOP) under good manufacturing practice (GMP) conditions and transplanted to the patients that required alveolar bone regeneration for the recovery of occlusal function [82].

Some case series using dental pulp micrografts in humans have been reported. The clinical studies by the group of Papaccio et al. were on the use of CD34-positive dental pulp cells combined with a collagen sponge to repair human mandible bone defects after extraction of third molars [83,84]. They found that regenerated tissue was composed of compact bone that was different from the alveolar bone. Aimetti et al. evaluated the potential clinical benefits of the application of dental pulp micrografts in the regenerative treatment of periodontal disease [85]. In this study, eleven chronic periodontitis patients presenting one deep intrabony defect and requiring extraction of one vital tooth were consecutively enrolled. They transplanted mechanical dissociative dental pulp that was filtered through 50-µm filters with a collagen sponge scaffold into the intrabony defect. Clinical and radiographic assessment revealed the effectiveness of the treatment. The clinical parameters of periodontal examination, clinical attachment level, and probing depth were improved and a remarkable stability of the gingival margin was observed. Bone regeneration was also confirmed by radiographic analysis. Besides this, a phase 1, open-label, single-blinded clinical trial has been planned to evaluate the safety and feasibility of an autologous DPSC therapy in patients with chronic disability after stroke [86]. A clinical trial to evaluate the safety and efficacy of dental pulp mesenchymal stem cells transplantation in patients with type 2 diabetes is also active and in the recruiting phase (NCT03658655)

## 4. Current Limitations and Perspectives

Although phenomena showing the effectiveness of DPSC transplantation therapy have been reported in various diseases, the mechanisms remain unclear. In our previous studies, the fate of transplanted MSCs was examined using a canine bone defect model [2]. GFP (green fluorescent protein)-expressing MSCs were present within the transplanted area, indicating that transplanted MSCs differentiated into osteoblasts and osteocytes and participated in bone regeneration. In this review, we mention several other studies that have also reported that DPSCs can differentiate into neural cells, dopaminergic-neurons, myotubes, hepatocyte-like cells, keratocytes, and RGC-like cells [27,28,30,31,32,33,34,35,36,47,56,65,71,73], and that the transplanted cells can migrate in the host tissues. The results of these studies indicate that the cells could participate in the regeneration of tissue. On the other hand, the paracrine effects of DPSCs have also been reported in many studies [4,48,68,69]. The cells might migrate to the injured region and stimulate recovery of function rather than replacement of damaged host tissues. The therapeutic benefits of DPSCs may be due to a combination of cell-autonomous and paracrine effects. Further studies are needed to clarify the mechanisms of the therapeutic properties of DPSCs.

As shown in Table 1, there have been many pre-clinical studies using animal disease models that have indicated the therapeutic abilities of DPSCs. However, only few clinical trials have been published so far. In order to verify the clinical utility of DPSCs and SHED, large-scale clinical trials in patients should be conducted. Major challenges to transplantation into clinical therapy still remain, such as strict regulations and the high cost of cell processing. The protocols used for the extraction/purification/expansion of these cells and the grafting approaches, including vehicles and scaffolds, were different in each previous study. As the relevant method was not established, it should also be developed for efficient treatment. That is to say, the optimization of cell processing and transplantation methods is also important: the way of preparing good-quality cells, transplantation cell numbers, a safety evaluation method, and so on. In addition, the limitations of autologous cells transplantation have been reported [87]. Preparation of a patient’s own stem cells for every patient is not efficient because of the time and cost required. Therefore, the utility of allogeneic transplantation and a stem cell banking system should be developed.

## 5. Conclusions

There are many kinds of stem cells, including pluripotent stem cells and adult stem cells. Pluripotent stem cells such as embryonic stem cells (ES cells) and induced pluripotent stem cells (iPS cells) are thought to be emerging tools in regenerative medicine, but there are a lot of problems, including ethical and safety concerns, that should be overcome before clinical use. Bone marrow-derived mesenchymal stem cells are the most widely studied and utilized adult stem cells in clinical settings so far. Recently, dental pulp has been found to be a potential alternative candidate stem cell source for clinical use, due to its high proliferative ability and being beneficial for cellular therapy in various applications. In addition, it can be obtained safety and easily from unnecessary teeth without significant morbidity or ethical concerns. As shown in this review, previous studies have indicated that DPSCs are useful for various diseases such as neurological disease, circulatory disease, diabetes, liver disease, eye disease, immune disease, and oral disease. Nevertheless, the challenge of understand the mechanisms underlying the therapeutic effects of DPSCs still remains before DPSC-based therapies can be translated into clinical application to patients. Further studies are needed to test the application of DPSCs in various therapies, but DPSCs have great potential to provide powerful tools for regenerative medicine.

## Figures and Tables

**Table 1:** Therapeutic potential of Dental pulp stem cells (DPSCs) in various diseases.

Disease	Cells	Study Model (Application Method)	Outcome	Ref.
**Spinal cord injury (SCI)**	Human DPSCs, SHED (stem cells from human exfoliated deciduous teeth)	Transplantation into a completely transected rat SCI model	DPSCs and SHED had neuroregenerative activities including inhibition of apoptosis of neurons, astrocytes, and oligodendrocytes, promoted the regeneration of transected axons, and replaced lost cells that fulfill many requirements for functional recovery after SCI.	[31]
	SHED, neural induced SHED (iSHED)	Injection into the spinal cord lesions of rat acute contused SCI model	Transplanted SHED and iSHED showed neuronal and glial differentiation and displayed significant locomotor functional recovery.	[34]
	Human DPSCs	Transplanted into completely transected rat spinal cord	DPSCs presented tissue regenerative capability after SCI through their immunomodulatory, differentiation, and protection capacity.	[35]
	SHED	Transplanted into completely injured rat spinal cord	The acute transplantation of SHED reduces early neuronal apoptosis, contributing to tissue and motor neuron preservation and hind limb functional recovery.	[36]
**Parkinson’s disease (PD)**	SHED	Intrastriatal transplantation of SHED and SHED-derived spheres in Parkinsonian rats	SHED and SHED-derived spheres differentiated into dopaminergic neurons and ameliorated behavioral impairment.	[37]
	SHED	Intrastriatal transplantation of SHED and SHED-derived dopaminergic neuron-like cells (dSHED) in Parkinsonian rats	Engrafted dSHEDs survived in the striatum of Parkinsonian rats, improved the dopamine level more efficiently than engrafted undifferentiated SHED, and promoted recovery from neurological deficits.	[38]
	Human DPSCs	MPP+ or rotenone-induced in vitro model of PD; indirect co-culture system with mesencephalic cell cultures	DPSCs showed a protective effect on dopamine neurons; dopamine uptake, and an increased number of spared tyrosinehydroxylase (TH)+ cells.	[39]
**Alzheimer’s disease (AD)**	Rat dental pulp cells	In vitro model of AD	The co-culture with dental pulp cells significantly attenuated 6-hydroxydopamine and Abeta(1-42)-induced toxicity in primary cultures of hippocampal neurons.	[40]
	Human DPSCs	In vitro model of AD established by okadaic acid (OA)-induced damage to human neuroblastoma cell line	DPSCs caused a significant increase in the viability and a decrease in apoptosis of AD model cells and phosphorylation at Ser 396 of Tau protein was significantly suppressed.	[41]
**Cerebral ischemia**	CD31^−^/CD146^−^ side population (SP) cells from porcine dental pulp	Transplanted into brain of middle cerebral artery occlusion rat	The cells promoted migration and differentiation of the endogenous neuronal progenitor cells, induced vasculogenesis, and ameliorated ischemic brain injury.	[42]
	Human DPSCs	Intracerebral transplantation of human DPSCs 24 h following focal cerebral ischemia in rat model	Human DPSC treatment enhanced functional recovery of post-stroke sensorimotor deficits.	[43]
	Human DPSCs	Intravenous transplantation of human DPSCs in a rat stroke model	DPSCs had a better effect on infarct size and significantly decreased reactive gliosis compared with bone-marrow-derived MSCs.	[44]
	Rat DPSCs	Intravenous administration in rat model of focal cerebral ischemia	DPSCs migrated into the boundary of ischemic areas and expressed neural specific markers, reducing infarct volume and cerebral edema.	[45]
**Myocardial Infarction**	Human DPSCs	Intramyocardial injection into nude rats with acute myocardial infarction	Cell-treated animals showed an improvement in cardiac function, observed by changes in anterior wall thickening, and a reduction in infarct size; angiogenesis was increased.	[46]
**Muscular dystrophy**	SHED	Systemic or intramuscular transplantation of SHED in golden retriever muscular dystrophy dogs	Donor SHED can engraft, differentiate, and persist in the host muscle. Systemic multiple deliveries seemed more effective than local injections.	[47]
	Human DPSCs	Intramuscular injection of pre-differentiated DPSCs into mdx/SCID mice, an immune-compromised animal model of Duchenne muscular dystrophy	DPSCs engrafted within the host muscle, promoted angiogenesis, and reduced fibrosis.	[48]
	Human dental pulp pluripotent-like stem cells (DPPSC)	DPPSC injection in dystrophic mice	DPPSC differentiated into both endothelial cells and smooth muscle cells and contributed to myogenic regeneration.	[49]
**Diabetes**	Human DPSCs, SHED	Transplantation of islet-like cell clusters (ICCs) derived from SHED into streptozotocin-induced diabetic mice	Mice transplanted with macro-capsules containing ICCs were restored to normoglycemia.	[50]
	Mouse DPSCs	Endovenous transplantation of DPSCs into streptozotocin-induced diabetes type 1 model	Transplanted DPSCs were confirmed in or surrounding pancreatic islets and DPSC transplantation improved pancreatic damage, renal function, and painful neuropathy.	[51]
	Rat DPSCs	Transplantation into the unilateral hind limb skeletal muscles of diabetic rat	DPSC transplantation improved sciatic nerve conduction velocity and sciatic nerve blood flow, ameliorated sural nerve axonal circularity, and decreased the macrophages in diabetic sciatic nerves.	[52]
	Human DPSCs	Intravenous or intramuscular transplantation of DPSCs into streptozotocin -induced neuropathic rats	DPSC transplantation through both routes was beneficial for the retrieval of neuropathic parameters of diabetic neuropathy.	[53]
**Liver disease**	SHED	Intrasplenic transplantation into the liver dysfunction of carbon tetrachloride-treated mice	Transplanted SHED directly transformed into hepatocytes, improved hepatic dysfunction, and led to anti-fibrotic and anti-inflammatory effects in the recipient livers.	[54]
	Human DPSCs	Transplantation of hepatically differentiated DPSCs into carbon tetrachloride-treated mice	The combination of melatonin and hDPSC significantly suppressed liver fibrosis and restored alanine transaminase, aspartate transaminase, and ammonia levels.	[55]
	Human DPSCs	Transplantation of hepatically differentiated DPSCs into carbon tetrachloride-treated mice	The combination of hDPSCs and PIN1 inhibitor juglone into CCl4-injured mice significantly suppressed liver fibrosis and restored serum levels of alanine transaminase, aspartate transaminase, and ammonia.	[56]
**Eye disease**	Human DPSCs	Keratocyte differentiated DPSCs were injected into mouse corneal stroma	Human DPSCs produced corneal stromal extracellular matrix and did not affect corneal transparency or induce immunological rejection.	[57]
	SHED sheet	Transplantation onto the corneal bed in rabbit model of limbal stem cell deficiency	Transplantation of a tissue-engineered human undifferentiated immature DPSC sheet was successful for the reconstruction of corneal epithelium.	[58]
	Human DPSCs	Contact lenses pre-seeded with DPSCs were transferred onto corneas	DPSCs transdifferentiated into corneal epithelial progenitors and established a barrier to the invasion of the cornea.	[59]
	Human DPSCs	Intravitreal transplantation in rodent model of glaucoma	DPSC provided significant protection from retinal ganglion cell (RGC) loss and preserved visual function.	[60]
**Immune disease**	SHED	Systemic transplantation to SLE-like murine model	SHED transplantation was capable of effectively reversing systemic lupus erythematosus (SLE)-associated disorders in SLE-like mice.	[61]
**Oral disease**	Canine CD105+ DPSCs	Transplantation into an adult canine model of pulpectomy	Complete pulp regeneration with neurogenesis and vasculogenesis occurred after transplantation of CD105+ DPSCs in pulpectomized root canals in canines.	[62]
	SHED	Injection into full-length human root canals model of pulpectomy	SHED survive and differentiate into odontoblasts when transplanted into full-length human root canals with injectable scaffolds.	[63]
	Canine DPSCs	Implantation into canine periodontitis model	Regeneration of cementum, bone, and periodontal ligament was observed.	[64]
	Canine DPSCs, deciduous tooth stem cells	Transplantation into canine alveolar bone atrophy model	Regeneration of well-formed mature bone was observed and dental implants were successfully installed in the regenerated bone.	[21]
